# Stability of Olmesartan Medoxomil Tablets in Unit-Dose Packaging With Metformin Hydrochloride Tablets

**DOI:** 10.7759/cureus.95626

**Published:** 2025-10-28

**Authors:** Ryuto Ohashi, Satoshi Yasunaga, Atsushi Ishimura, Takahito Furuya, Yuki Aizawa, Takayuki Omori, Takuro Kurita, Takanori Nakajima, Yusuke Takizawa

**Affiliations:** 1 Division of Clinical Pharmaceutics, Faculty of Pharmaceutical Sciences, Nihon Pharmaceutical University, Saitama, JPN; 2 Laboratory of Clinical Pharmacy Assessment, Hoshi University, Shinagawa, JPN; 3 Department of Pharmacy, Juntendo University Hospital, Tokyo, JPN; 4 Division of Practical Pharmaceutical Sciences, Faculty of Pharmaceutical Sciences, Nihon Pharmaceutical University, Saitama, JPN

**Keywords:** discoloration, drug interaction, drug stability, formulation design, generic drugs, metformin hydrochloride, olmesartan medoxomil, patient adherence, pharmaceutical excipient, unit-dose packaging

## Abstract

Background: The widespread implementation of unit-dose packaging has improved medication adherence, particularly in elderly populations. However, potential physicochemical interactions between co-packaged drugs remain a concern. Therefore, the present study evaluated the stability of metformin hydrochloride (MET) tablets and olmesartan medoxomil (OLMMD) tablets (orally disintegrating tablet: OLMOD/standard tablet: OLM), including both brand name and generic products, when unit-dose packaged and stored under a high temperature and humidity (40°C, 75% RH) or room temperature for up to 28 days.

Methods: A visual inspection and HPLC quantification were used to assess the discoloration of MET and content of OLMMD.

Results: When MET1 (brand name) or MET2 (generic) was packaged with OLMOD1 (brand name), no discoloration or degradation was observed. However, with MET3 (generic), time-dependent pink discoloration occurred when co-packaged with OLMOD2 or OLMOD3 (generics). OLMMD content remained nearly 100% even in formulations inducing MET discoloration. Differences were attributed to excipient and manufacturing variability. Notably, OLMOD1 uniquely contained β-cyclodextrin, which may stabilize OLMMD via inclusion complexation. By contrast, MET discoloration may reflect hygroscopicity and variability in film-coating properties. Although pharmacological efficacy was unaffected due to preserved OLMMD stability and the harmless nature of MET discoloration, visual changes may reduce patient adherence. Thus, selecting stable combinations that avoid discoloration is important in clinical practice, especially for elderly or cognitively impaired patients.

Conclusions: This study highlights the importance of excipient selection and formulation design in ensuring stability in unit-dose packaging. The development of compatibility guidelines and formulation reference lists is warranted to support safe co-packaging practices.

## Introduction

Recent global increases in elderly populations and patients with polypharmacy have led to the widespread adoption of unit-dose packaging as a part of medication adherence support systems. Unit-dose packaging, which involves repackaging medications into individualized doses according to administration times, originated in hospitals in the United States in the late 1950s and early 1960s as a means of improving medication safety and reducing dispensing errors [1,2]. The system gained traction in Europe by the 1980s and was introduced in Japan in the late 1970s, particularly within home care and geriatric settings [3]. Unit-dose dispensing is now commonly practiced in community pharmacies in Japan and other Asian countries and has contributed to improvements in adherence and reductions in medication errors [4,5].

Despite these benefits, unit-dose packaging presents challenges related to the physical and chemical stabilities of co-packaged drugs. Some drug combinations may undergo physicochemical interactions when stored together, potentially compromising their stability, efficacy, or safety [6]. Therefore, it is essential to evaluate the compatibility of and potential interactions between pharmaceutical formulations when implementing unit-dose packaging strategies, particularly when involving moisture-sensitive or chemically reactive compounds.

Among oral antidiabetic agents, metformin hydrochloride (MET), a biguanide-class drug, is known for its high hygroscopicity. When unit-dose packaged together with olmesartan medoxomil (OLMMD), an angiotensin II receptor blocker widely used as an antihypertensive, visible discoloration, particularly the pink coloration of metformin tablets, has been reported [7]. This phenomenon is attributed to a chemical reaction known as the Voges-Proskauer (VP) reaction. In this reaction, the (5-methyl-2-oxo-1,3-dioxol-4-yl)methyl group (DMDO group) released from hydrolyzed OLMMD is converted into diacetyl and acetoin. These intermediates subsequently react with the guanidino group of metformin, resulting in the formation of a pink-colored complex [8].

This reaction is considered to occur under elevated temperatures, the presence of sufficient moisture, and a confined environment, such as that created by unit-dose packaging. Although the discoloration itself is generally considered to not directly affect the pharmacological activities of drugs, it may be perceived by patients as a sign of deterioration, potentially leading to the discontinuation of therapy. This makes it a practically significant issue in pharmaceutical care. OLMMD, a widely prescribed antihypertensive agent, is often co-packaged with other medications in unit-dose formats; however, its physicochemical stability when packaged with MET has not been thoroughly investigated.

The oral bioavailability of MET was previously reported to be approximately 60.6% [9]. If acetylation occurs via the aforementioned VP reaction, it is conceivable that the resulting increase in lipophilicity may enhance membrane permeability, thereby potentially increasing the absorption rate of metformin. This raises concerns about elevated plasma concentrations of metformin and the associated risk of adverse effects. Although some studies suggested that this color change did not impact the pharmacological efficacy of the drug [10], it remains unclear whether these chemical modifications affect the incidence of adverse effects.

On the other hand, the bioavailability of OLMMD is approximately 25.6% [11]. OLMMD is a prodrug designed to increase the membrane permeability of its active metabolite, olmesartan (OLM), by incorporating a medoxomil group, which is hydrolyzed in the gastrointestinal tract to release OLM, diacetyl, and carbon dioxide. Therefore, if unit-dose packaging conditions promote the premature hydrolysis of the medoxomil moiety, leading to the conversion of OLMMD into OLM prior to administration, the resulting decrease in lipophilicity may reduce membrane permeability and ultimately diminish pharmacological efficacy.

Therefore, the aim of the present study was to quantitatively evaluate not only the color change in MET tablets, but also the degradation of OLMMD when co-packaged in a unit-dose form with OLMMD tablets. The present results are expected to provide valuable insights not only into the compatibility of OLMMD and MET during unit-dose packaging, but also into the potential interactions between OLMMD and other drugs containing guanidino groups. Furthermore, these results may serve as important foundational data for raising awareness of physicochemical changes caused by unit-dose packaging, thereby offering clinically meaningful information for safe and effective dispensing practices.

## Materials and methods

Medicine

MET tablets included one brand-name product (MET1) and two generic products (MET2 and MET3), each containing 250 mg of MET as labeled. Standard tablets and orally disintegrating (OD) tablets were both used for OLMMD. Since the brand-name version of the standard tablet has already been discontinued in Japan, two generic products (OLM1 and OLM2) were selected, each containing 10 mg of OLMMD as labeled. One brand-name product (OLMOD1) and two generic products (OLMOD2 and OLMOD3) were used for OD tablets, also containing 10 mg of OLMMD as labeled. All reagents were of analytical grade or higher.

Packaging conditions (temperature, humidity, and duration)

In unit-dose packaging, TEX Diamat (cellophane/polyethylene-laminated packaging paper; 70TD20M, Takazono Co., Ltd., Japan) was used to package MET and olmesartan OD or standard tablets (OLMOD/OLM) under multiple conditions. After packaging, the samples were stored under two conditions: at room temperature in the dark and at 40°C with 75% relative humidity. On days 7, 14, and 28 after packaging, the color change in MET was evaluated visually, and the content of OLMMD in OLMOD/OLM tablets was quantified using HPLC.

Visual color assessment of MET tablets

After the designated number of days (seven, 14, or 28 days) had elapsed, a visual inspection was conducted to assess colorimetric changes, followed by photographic documentation.

Assessment of the OLMMD content in OLMMD tablets

A stock solution of ethyl p-hydroxybenzoate (internal standard) was prepared by dissolving 1000 mg in a 1-L volumetric flask using an acetonitrile:water mixture (3:2, v/v) as the solvent. Regarding sample preparation, one tablet of the formulation was accurately weighed, and 5 mL of the acetonitrile:water mixture (3:2, v/v) was added. Then, V mL of the internal standard solution was added, and the total volume was adjusted to 10 mL using the same solvent. The mixture was sonicated for 10 minutes with intermittent shaking. Approximately 0.2 mg of OLMMD was added by incorporating an appropriate volume of the acetonitrile:water mixture to achieve a final volume of V mL, resulting in a solution with a concentration of 0.2 mg/mL. The solution was then centrifuged, and 5 mL of the supernatant was transferred to a new container. This aliquot was diluted to a final volume of 25 mL with the acetonitrile:water mixture (3:2, v/v), yielding the final sample solution. 

Measurements of OLMMD by HPLC

The measurement and analysis methods for OLMMD were performed by HPLC as follows. OLMMD was measured by reverse-phase HPLC using a Imtakt Cadenza 5CD-C18 column (150 × 4.0 mm, 5 μm) at 40°C. The mobile phase consisted of a mixture of 65% solution A (H₂O:HClO₄:NaClO₄ = 1000:1:5 g; v/v) and 35% solution B (100% acetonitrile), delivered at a flow rate of 1.0 mL/min. The injection volume was 10 µL, and detection was performed at 265 nm.

Statistical analysis

All results are expressed as the mean ± standard deviation (SD). The significance of differences between groups was analyzed using the unpaired Student’s *t*-test, and P < 0.05 was considered to be significant.

## Results

Physicochemical changes during the unit-dose packaging of brand-name OLMMD OD tablets with brand-name MET tablets

**Figure 1:**
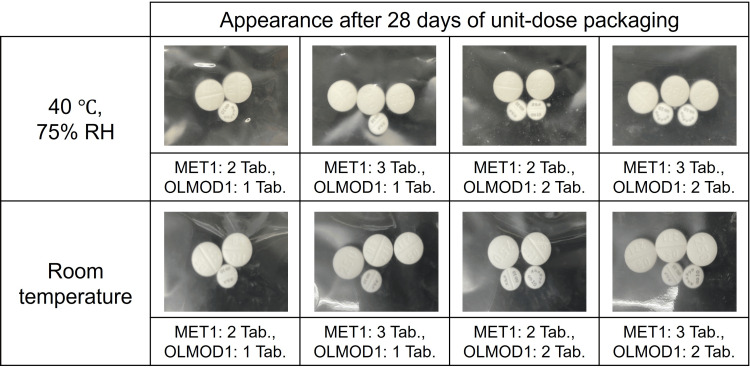
Color change of metformin hydrochloride tablets (MET1: brand-name product) after co-packaging (unit-dose packaging) with olmesartan medoxomil orally disintegrating tablets (OLMOD1: brand-name product). Abbreviations: MET, metformin hydrochloride tablet; OLMOD, olmesartan medoxomil orally disintegrating tablet; RH, relative humidity; Tab., tablet Storage conditions: 40°C and 75% RH or room temperature for 28 days Visual appearance was compared with freshly opened (unexposed) tablets used as controls.

The visual color change in the brand-name MET tablet (MET1) was initially evaluated after unit-dose packaging with the brand-name OLMMD OD tablet (OLMOD1) using various tablet count combinations. No visible color change was observed in MET1 after seven, 14, or 28 days under either storage condition, namely, room temperature in the dark or 40°C with 75% relative humidity (data for days 7 and 14 are not shown; data for day 28 are presented in Fig. 1).

**Figure 2:**
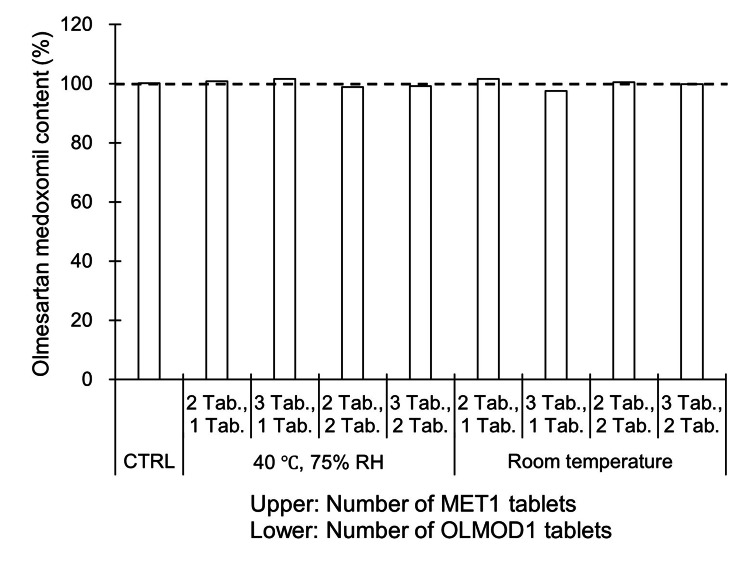
Content of olmesartan medoxomil in olmesartan medoxomil orally disintegrating tablets (OLMOD1: brand-name product) after 28 days of co-packaging (unit-dose packaging) with metformin hydrochloride tablets (MET1: brand-name product). Abbreviations: MET, metformin hydrochloride tablet; OLMOD, olmesartan medoxomil orally disintegrating tablet; RH, relative humidity; Tab., tablet; CTRL, control Storage conditions: 40 °C and 75% RH or room temperature for 28 days Control (CTRL) represents freshly opened (unexposed) tablets. *n *= 1 indicates that the data represent one independent sample obtained from a single manufacturing lot; comparable results were confirmed using other lots (data not shown). Because data were obtained from different lots, no statistical analysis was performed.

In addition, the OLMMD content in OLMOD1 was quantified, and no significant change was noted under any of the conditions tested (data for days 7 and 14 are not shown; data for day 28 are presented in Fig. 2).

Physicochemical changes during the unit-dose packaging of brand-name OMMMD OD tablets with generic MET tablets

**Figure 3:**
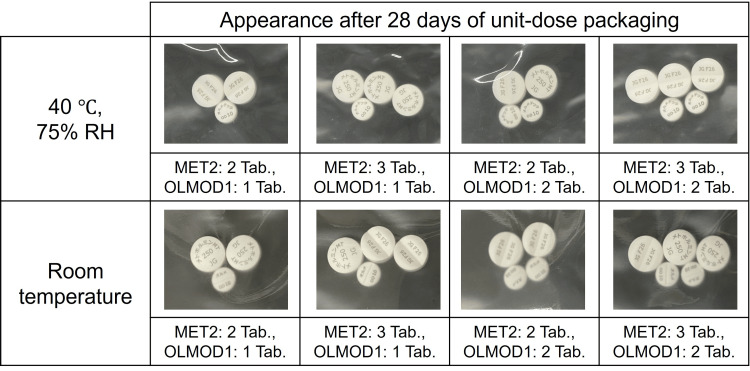
Color change of metformin hydrochloride tablets (MET2: generic product) after co-packaging (unit-dose packaging) with olmesartan medoxomil orally disintegrating tablets (OLMOD1: brand-name product). Abbreviations: MET, metformin hydrochloride tablet; OLMOD, olmesartan medoxomil orally disintegrating tablet; RH, relative humidity; Tab., tablet Storage conditions: 40 °C and 75% RH or room temperature for 28 days Visual appearance was compared with freshly opened (unexposed) tablets used as controls.

The MET tablet was replaced with a generic product (MET2), and unit-dose packaging was performed with the brand-name OLMMD OD tablet (OLMOD1) under the same conditions as those shown in Fig. 1. Similar to the results with MET1 and OLMOD1, no visible color change was observed in MET2 after seven, 14, or 28 days under either storage condition, namely, room temperature in the dark or 40°C with 75% relative humidity (data for days 7 and 14 are not shown; data for day 28 are presented in Fig. 3).

**Figure 4:**
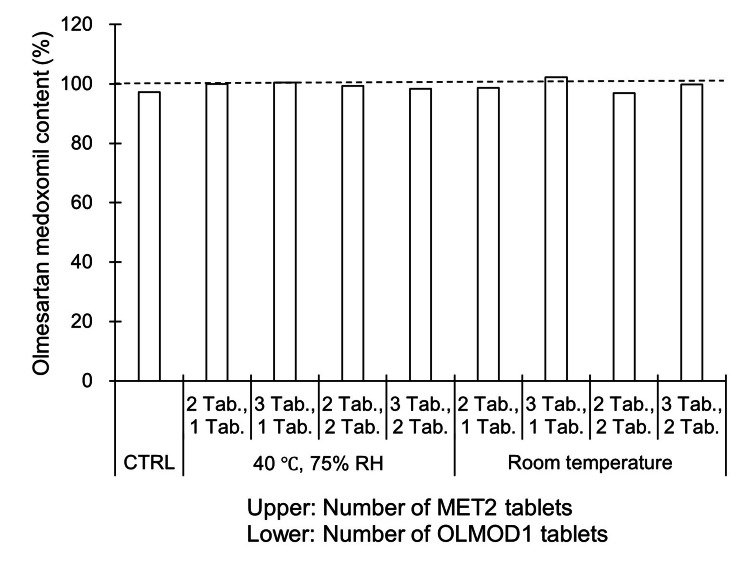
Content of olmesartan medoxomil in olmesartan medoxomil orally disintegrating tablets (OLMOD1: brand-name product) after 28 days of co-packaging (unit-dose packaging) with metformin hydrochloride tablets (MET2: generic product). Abbreviations: MET, metformin hydrochloride tablet; OLMOD, olmesartan medoxomil orally disintegrating tablet; RH, relative humidity; Tab., tablet; CTRL, control Storage conditions: 40 °C and 75% RH or room temperature for 28 days Control (CTRL) represents freshly opened (unexposed) tablets. *n *= 1 indicates that the data represent one independent sample obtained from a single manufacturing lot; comparable results were confirmed using other lots (data not shown). Because data were obtained from different lots, no statistical analysis was performed.

Furthermore, in the combination of MET2 and OLMOD1, no significant change was detected in the OLMMD content in OLMOD1 under any of the conditions tested (data for days 7 and 14 are not shown; data for day 28 are presented in Fig. 4).

Physicochemical changes during the unit-dose packaging of generic OLMMD OD tablets with generic MET tablets

The effects of unit-dose packaging using only generic products were investigated. Instead of MET2 (used in Fig. 2), a different generic MET tablet (MET3) was used. Two generic standard tablets (OLM1 and OLM2) and three OD tablets (OLMOD1: brand-name, OLMOD2 and OLMOD3: generics) were used for OMMMD. Storage was conducted under the conditions of 40°C and 75% relative humidity only. 

**Figure 5:**
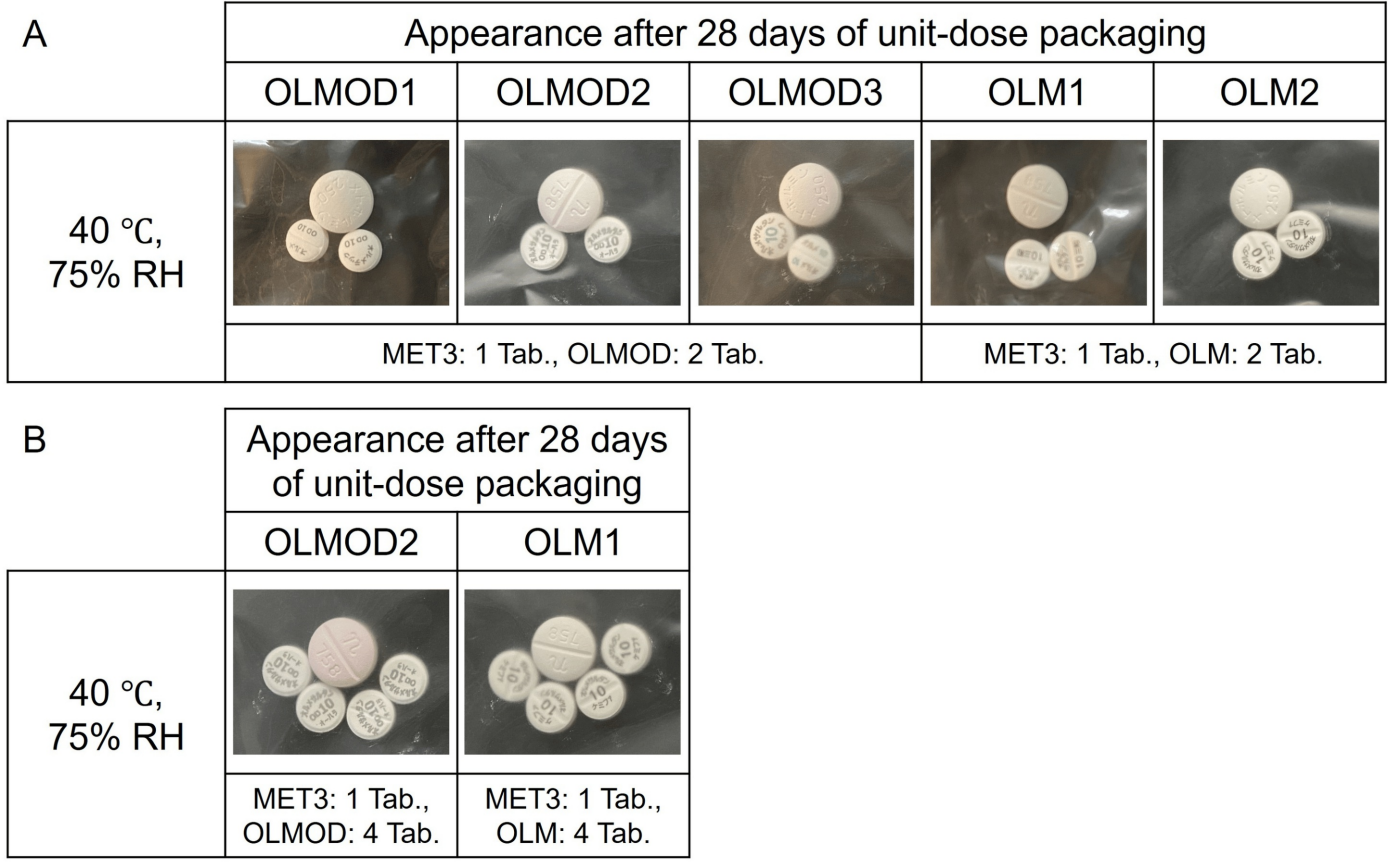
Color change of metformin hydrochloride tablets (MET3: generic product) after co-packaging (unit-dose packaging) with olmesartan medoxomil orally disintegrating tablets (OLMOD1: brand-name product, OLMOD2 and OLMOD3: generic products) or olmesartan medoxomil standard tablets (OLM1 and OLM2: generic products). Abbreviations: MET, metformin hydrochloride tablet; OLMOD, olmesartan medoxomil orally disintegrating tablet; OLM, olmesartan medoxomil standard tablet; RH, relative humidity; Tab., tablet Panel A: one MET packaged with two OLMOD or OLM. Panel B: one MET packaged with four OLMOD or OLM. Storage conditions: 40 °C and 75% RH for 28 days Visual appearance was compared with freshly opened (unexposed) tablets used as controls.

The time-dependent pink discoloration of MET3 was observed when co-packaged with OLMOD2 or OLMOD3 (data for days 7 and 14 are not shown; data for day 28 are presented in Fig. 5A). Furthermore, when one tablet of MET3 was packaged with four tablets of OLMOD2, a combination in which MET3 showed pronounced discoloration, the extent of pink coloration increased further. In contrast, when the same packaging conditions (MET3 1 tablet, OLMOD2 4 tablets) were applied with OLM1 (which had no effect on the color of MET3), no discoloration was observed in MET3 (data for days 7 and 14 are not shown; data for day 28 are presented in Fig. 5B).

**Figure 6:**
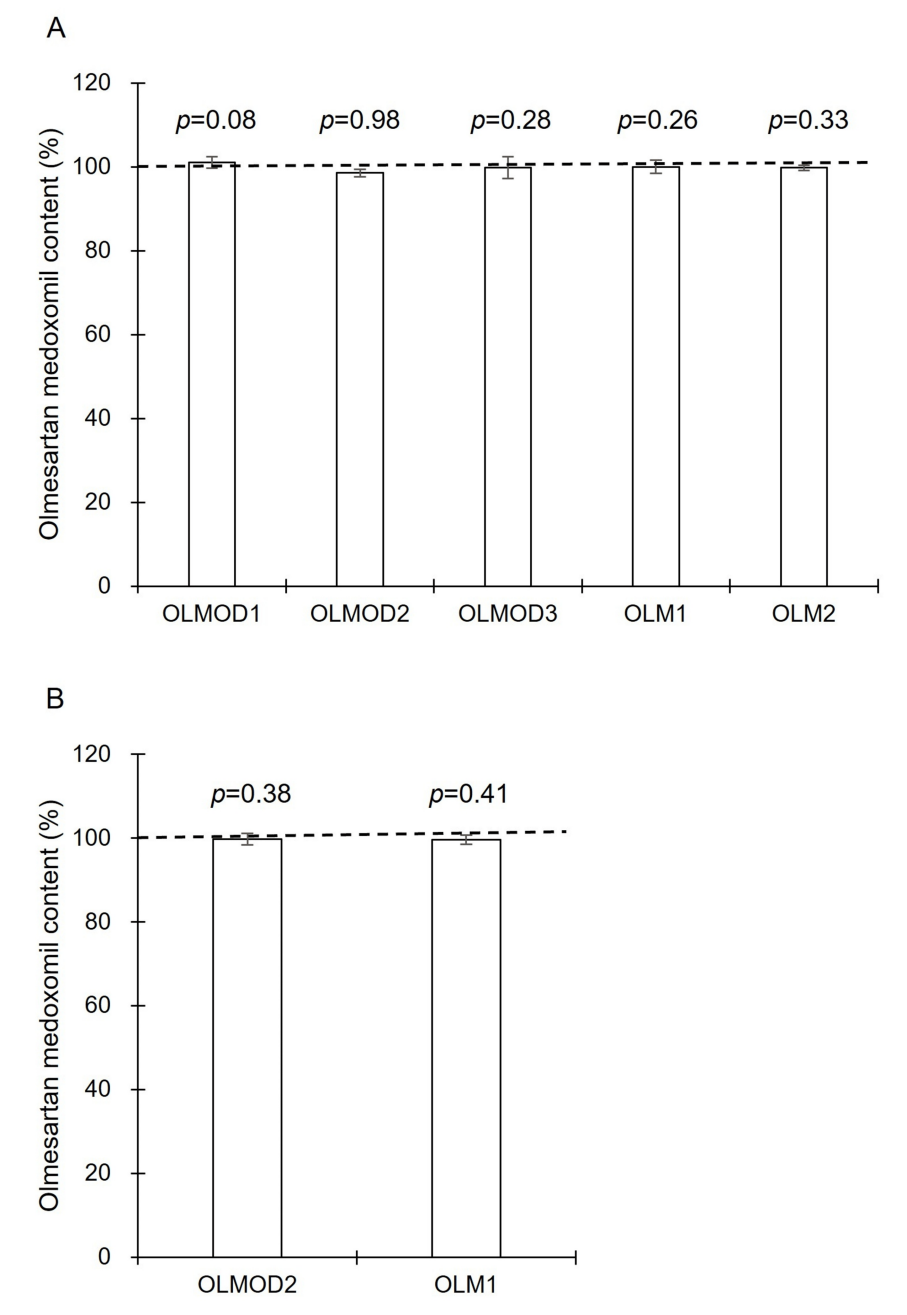
Content of olmesartan medoxomil in olmesartan medoxomil orally disintegrating tablets (OLMOD1: brand-name product, OLMOD2 and OLMOD3: generic products) or olmesartan medoxomil standard tablets (OLM1 and OLM2: generic products) after 28 days of co-packaging (unit-dose packaging) with metformin hydrochloride tablets (MET3: generic product). Abbreviations: MET, metformin hydrochloride tablet; OLMOD, olmesartan medoxomil orally disintegrating tablet; OLM, olmesartan medoxomil standard tablet; RH, relative humidity Panel A: one MET packaged with two OLMOD or OLM. Panel B: one MET packaged with four OLMOD or OLM. Storage conditions: 40 °C and 75% RH for 28 days Data represent mean ± SD of four independent samples obtained from the same lot (*n* = 4). The olmesartan medoxomil content (%) was calculated as the ratio of the measured amount of olmesartan medoxomil in each tablet—either freshly opened or after 28 days of co-packaging—to the nominal labeled amount. Statistical comparisons were performed between freshly opened and co-packaged tablets for each product using a two-tailed Student’s *t*-test; exact *p* values are shown in the figure.

In addition, as shown in Fig. 2 and Fig. 4, a quantitative analysis of OLMMD in OLMOD and OLM after packaging was performed. No statistically significant decrease in the content of OLMMD was observed under any condition, including those involving OLMOD2 and OLMOD3, which caused the discoloration of MET3 (data for days 7 and 14 are not shown; data for day 28 are presented in Figs. 6A, 6B). Although a slight, non-significant reduction was noted in some formulations, the overall OLMMD content remained close to the labeled amount throughout storage.

## Discussion

In the present study, unit-dose packaging was performed using three types of MET tablets (one brand name and two generics), three types of OLMMD OD tablets (one brand name and two generics), and two types of OLMMD standard tablets (both generics). We investigated the presence or absence of discoloration and the stability of the active pharmaceutical ingredients after storage under high temperature and humidity conditions (40 °C, 75% RH) and at room temperature.

The effects of unit-dose packaging between the two brand-name products, MET1 and OLMOD1, were initially investigated. Various tablet count combinations of MET1 and OLMOD1 were tested, and no discoloration of MET1 was observed up to 28 days after packaging, even under high temperature and humidity conditions (Fig. 1). Furthermore, no change was observed in the content of OLMMD in OLMOD1 (Fig. 2). These results suggest that the unit-dose packaging of MET1 and OLMOD1 did not result in the discoloration of metformin or any significant degradation of OLMMD, indicating that the co-packaging of these two brand-name products is appropriate.

The effects of unit-dose packaging using a generic metformin tablet (MET2) with OLMOD1 were then examined. Similar to the results shown in Fig. 1 and Fig. 2, no discoloration of MET2 was observed up to 28 days under high temperature and humidity conditions (Fig. 3), and no significant change in the OLMMD content in OLMOD1 was detected (Fig. 4). These results suggest that the absence of discoloration in MET tablets was attributed to the high stability of OLMOD1 during unit-dose packaging and the minimal, non-significant degradation of OLMMD.

Therefore, further investigations were conducted using other OLMOD and OLM tablets. A different generic MET tablet (MET3) was used to evaluate unit-dose packaging with each OLMOD and OLM product. Similar to MET1 and MET2, no discoloration of MET3 was observed when co-packaged with the brand name OLMOD1. However, the time-dependent pink discoloration of MET3 was observed when co-packaged with generic OLMOD2 and OLMOD3 (Fig. 5A). By contrast, no discoloration of MET3 was noted when co-packaged with the generic standard tablets OLM1 and OLM2 (Fig. 5A). Furthermore, when the number of OLMOD or OLM tablets co-packaged with MET3 was increased, the pink discoloration of MET3 became more pronounced with OLMOD2, while no discoloration was observed with OLM1 under the same conditions. These results suggest significant differences in the degree of OLMMD degradation among different OLMMD formulations. Interestingly, despite the apparent progression of the reaction leading to the discoloration of MET3 when co-packaged with OLMOD2 and OLMOD3the quantified content of OLMMD after 28 days remained close to 100%, with no statistically significant reduction detected. This was similar to the content levels in OLMOD1, OLM1, and OLM2, which did not induce any discoloration in MET3.

In the present study, the quantification of OLMMD was performed using sample solutions prepared by dissolving the tablet in 50 mL of solvent. Since each OLMOD/OLM tablet used in the present study contained 10 mg of OLMMD, it was confirmed that no significant degradation occurred in OLMOD2 and OLMOD3, even those that caused the visible discoloration of MET3, at a concentration of 10 mg/50 mL. In clinical practice, it is standard for conventional tablets to be taken with at least 50 mL of water. Even in the case of OD tablets, which are designed to be taken without water, the environment in the stomach after ingestion is expected to provide a sufficiently large volume to simulate dilution. Therefore, it is unlikely that any degradation of OLMMD potentially occurring during unit-dose packaging had a significant impact on its pharmacological efficacy in vivo. Collectively, these results suggest that the pharmacological activity of OLM was not compromised by co-packaging with MET tablets under the conditions tested.

In the present study, the effects of various OLMOD/OLM tablets on MET1 and MET2 were not individually assessed; therefore, it remains inconclusive whether some MET tablets are more or less prone to color changes upon unit-dose packaging with OLMOD/OLM. However, as shown in Table 1, the types of pharmaceutical excipients contained in the MET formulations used in the present study markedly varied, suggesting that the sensitivity of each formulation to co-packaging conditions may differ. Metformin is highly hygroscopic and, thus, moisture is absorbed into the tablet matrix under high humidity conditions [12]. Differences in excipients, manufacturing processes, and coating technologies may lead to variations in the stability of the tablets during unit-dose packaging [13]. Differences in the type, thickness, and hydrophilicity of film coatings may affect the extent of moisture uptake and the degree to which interactions with other drugs are suppressed. These factors may contribute to the observed differences in color changes among formulations.

**Table 1:** Pharmaceutical excipients in each MET tablet Symbols: ○ = present; – = absent. Each MET contained 250 mg of metformin hydrochloride as labeled. Product names are anonymized in accordance with journal policy. Abbreviations: MET, metformin hydrochloride tablet

Pharmaceutical excipients	MET1	MET2	MET3
Povidone	〇	-	-
Magnesium stearate	〇	〇	〇
Hydroxypropylmethyl cellulose	〇	〇	〇
Macrogol 400	〇	-	-
Macrogol 6000	〇	〇	-
Talc	〇	〇	〇
Light anhydrous silicic acid	-	〇	〇
Titanium oxide	-	〇	〇
Hydroxypropyl cellulose	-	〇	〇
Carnauba wax	-	〇	-

By contrast, the degree of degradation observed in the OLMOD/OLM formulations appeared to differ among products. Table 2 summarizes the pharmaceutical excipients contained in the OLMOD and OLM tablets used in this study. The composition of excipients significantly varied among the three OLMOD formulations. Several excipients were unique to OLMOD1, among which β-cyclodextrin may have contributed to suppressing the degradation of OLMMD. Since OD tablets generally exhibit higher disintegration rates than conventional tablets, the risk of OLMMD degradation may increase accordingly. However, the inclusion complexation effect of β-cyclodextrin, present only in OLMOD1, may have stabilized OLMMD during storage [14,15]. This suggests that β-cyclodextrin may contribute to the apparent stability of OLMOD1 by preventing oxidative degradation; however, this interpretation remains speculative and requires further mechanistic confirmation through dedicated physicochemical analyses. Although β-cyclodextrin was not present in the standard OLM tablets (OLM1 and OLM2), these formulations may offer greater stability due to their lower disintegration capacity than OD tablets. Furthermore, since OLM1 and OLM2 share the same excipient composition and neither induced the discoloration of MET tablets, pharmaceutical excipients and manufacturing processes may significantly affect the degradation stability of OLMMD. Our research group previously reported that differences in excipients may affect the dissolution behavior [16] and membrane permeability [17-19] of various formulations. These findings suggest that, in addition to β-cyclodextrin, other excipients may be involved in the physicochemical stability of OLMMD during unit-dose packaging.

**Table 2:** Pharmaceutical excipients in each OLMOD/OLM tablet Symbols: ○ = present; – = absent. Each OLM and OLMOD contained 10 mg of olmesartan medoxomil as labeled. Product names are anonymized in accordance with journal policy. Abbreviations: OLMOD, olmesartan medoxomil orally disintegrating tablet; OLM, olmesartan medoxomil standard tablet

Pharmaceutical excipients	OLMOD1	OLMOD2	OLMOD3	OLM1	OLM2
Crystalline cellulose	〇	〇	-	〇	〇
β-cyclodextrin	〇	-	-	-	-
Carboxymethyl cellulose	〇	-	-	-	-
Sucralose	〇	-	-	-	-
Acesulfame potassium	〇	-	-	-	-
Magnesium stearate	〇	〇	-	〇	〇
Yellow ferric oxide	〇	-	-	-	-
Flavoring agent	〇	-	〇	-	-
dl-α-tocopherol	〇	-	-	-	-
D-mannitol	-	〇	〇	-	-
Low-substituted hydroxypropylcellulose	-	〇	-	〇	〇
Corn starch	-	〇	-	-	-
Hydroxypropyl cellulose	-	〇	〇	〇	〇
Light anhydrous silicic acid	-	〇	〇	-	-
Aspartame	-	〇	〇	-	-
l-menthol	-	〇	-	-	-
Lactose hydrate	-	-	〇	〇	〇
Titanium oxide	-	-	〇	-	-
Stearic acid	-	-	-	〇	〇
Four other ingredients	-	-	〇	-	-

Nevertheless, although the discoloration of MET was observed in some combinations, no significant visual changes or decreases in the content of the active pharmaceutical ingredient, OLMMD, were detected in any of the OLMOD/OLM formulations. This may be attributed to the relatively high stability of OLMMD as a prodrug, which is less prone to degradation via hydrolysis or oxidation. In addition, the stability of OLMMD may be maintained by a number of formulation factors, such as coating technologies and the design of excipients. OLMMD remained chemically stable even after being co-packaged with MET in the present study, which provides important evidence to show that the chemical interaction between these two drugs during unit-dose packaging was minimal.

From a pharmacological perspective, if the active ingredient in OLMOD/OLM remains quantitatively stable after storage and the discoloration of MET is limited to visual changes without affecting its efficacy, theoretically there is no major impact on the overall therapeutic effect. Previous studies indicated that the discoloration of MET tablets did not compromise their pharmacological efficacy [10]. However, it is important to consider that patients may notice color changes during administration, which may lead to anxiety and the potential discontinuation of therapy. Therefore, even if the discoloration itself is clinically harmless, it may still pose a significant risk for reduced medication adherence. Therefore, even if there is no significant issue regarding the pharmacological efficacy of co-packaging the two drugs, the selection of combinations that do not result in discoloration represents a practical solution from the perspective of maintaining the physical appearance and stability of the formulation. This consideration is particularly crucial for elderly patients or those with cognitive impairments, for whom the visual appearance of medications may have a marked impact on daily adherence behavior. Therefore, ensuring the stability of the formulation is essential for supporting safe and sustained medication adherence in clinical practice.

While this study confirmed that the co-packaging of MET tablets (MET) and OLMMD tablets (OLMOD/OLM) did not significantly impact the stability of active pharmaceutical ingredients, the visible discoloration of MET tablets in some combinations may have practical consequences. In clinical settings, changes in the appearance of medications, particularly discoloration, may lead to confusion, mistrust, and even non-adherence, particularly among older patients or those with cognitive impairment. Previous studies demonstrated that patients were highly sensitive to the visual consistency of their medications, and may discontinue therapy when changes are perceived, even if these changes are clinically insignificant [20]. Therefore, even if pharmacological efficacy remains unaffected, avoiding combinations that lead to visual changes may be important from the perspective of medication adherence and patient safety.

The present study also highlights the variability in physical changes across generic products. Since different combinations of generics did or did not cause discoloration, this result underlines the need for more careful consideration when selecting formulations for unit-dose packaging. Further investigations are warranted to examine patient-reported outcomes related to visual drug changes, and to establish whether these changes correlate with adherence rates in real-world practice. In addition, expanding the evaluation to include in vitro tests, such as dissolution and permeability assays, may provide more detailed insights into formulation stability and the interchangeability of co-packaged generics.

This study has several limitations. First, the number of products and lots tested was limited. Although comparable trends were confirmed using additional lots, further studies with a larger sample size are warranted to confirm reproducibility. Second, the experiments were conducted under accelerated stress conditions (40 °C and 75% RH) to evaluate the potential for color change and degradation; therefore, the results may not fully represent typical storage conditions in clinical practice. Third, only visual appearance and drug content were evaluated, and more detailed analyses - such as chromatographic identification of degradation products or film-coating integrity testing-were not performed. Finally, this was an in vitro stability assessment without clinical outcome evaluation, and the findings should therefore be interpreted as preliminary and hypothesis-generating rather than confirmatory. In addition, visual assessment was performed by subjective observation without quantitative colorimetric analysis, which may limit the objectivity and reproducibility of the visual findings.

## Conclusions

The present study demonstrated that the stability of the unit-dose packaging of MET tablets and OLM/OLMMD tablets varied depending on the specific combination of formulations. Although no significant impact on pharmacological efficacy was observed, visible changes in tablet appearance, such as discoloration, were identified as a practical concern in clinical settings. These results highlight the need for future efforts to establish guidelines for the rapid assessment of discoloration risk, as well as the development of formulation selection lists based on stability in unit-dose packaging. Furthermore, pharmaceutical manufacturers need to pursue formulation designs that are optimized for compatibility and stability during co-packaging.
